# On Some of the Principal Effects Resulting from the Detachment of Fibrinous Deposits from the Interior of the Heart, and Their Mixture with the Circulating Blood

**Published:** 1853

**Authors:** Wm. Senhouse Kirkes

**Affiliations:** Licentiate of the Royal College of Physicians, &c.


*On some of the principal effects resulting from the Detachment of Fibrinous Deposits from the Interior of the Heart, and their mixture with the circulating blood.* By Wm. Senhouse Kirkes, M. D., Licentiate of the Royal College of Physicians, &c. Communicated by George Burrows, M. D., F. R. S., Physician to St. Bartholomew's Hospital.

That the fibrinous principle of the blood may, under certain circumstances, separate from the circulating fluid during life, and be deposited within the vascular system, especially on the valves of the heart, is a fact so clearly established, and so generally admitted, that I need only, at the outset of the communication I have the honor to present to this society, allude to it as a settled truth, and refer, for the proofs, to the various general works on diseases of the heart and blood-vessels, and to such special essays on the subject as those of Dr. Burrows* and Dr. Hughes.† From these sources may also be gathered nearly all that is yet known respecting the various conditions under which the deposition of fibrine takes place, and the several forms which the deposits assume. Into these general details I do not purpose entering, my object being simply to consider the effects which the deposits may produce on the system at large. It may, however, be premised that the forms of fibrinous concretions, to which my observations chiefly apply, are, first, the masses usually described as Lænnec's globular excrescenses; and, secondly, the granular or warty growths adhering to the valves and presenting innumerable varieties, from mere granules to large irregular fungous or cauliflower excrescences projecting into the cavaties of the heart.

Avoiding all discussion concerning these latter growths, I proceed at once to state that in whatever way they may

---

* Medical Gazette, vol. xvi, 1834–5.
† Guy's Hospital Reports, vol. iv, 1839.

originate, they are, when once formed, full of peril, and often remain so, even long after the circumstances which gave rise to them have passed by. If of large size and only loosely adherent, as they often are, one or more masses of even considerable magnitude may, at any time, be detached from the valves and conveyed with the circulating blood, until arrested within some arterial canal which may be completely plugged up by it, and thus the supply of blood to an important part be suddenly cut off, and serious, even fatal results ensue. Or, the deposits on the valves may be detached in smaller masses, and pass on into arteries of much less size, or even into the capillaries, where, being arrested, they may cause congestion, followed by stagnation and coagulation of the blood, with all the subsequent changes which blood coagulated with the living body is liable to undergo. In this way are probably induced many singular morbid appearances often observed in internal organs, and rarely well accounted for. Again, the masses of fibrine may soften, break up, and discharge the finely granular material resulting from their disintegration; and this, mingling and circulating with the blood, may give rise to various disturbances indicative of a contaminated state of this fluid, producing symptoms very similar to those observed in phlebitis, typhus, and other analogous blood diseases. In one or more of these several ways, and probably in others not yet clearly recognized, fibrinous material detached from the valves, or any other part of the interior of the heart, may be the cause of serious secondary mischief in the body.

It appears unnecessary to insist here on the possibility of any of the various forms of fibrinous deposit found within the heart, being detached either spontaneously or by the mere force with which the current of blood passes over the surfaces on which they are placed. For it is well known, that after death a very gentle force, sometimes even the slightest touch, will loosen and dislodge both small granular particles and masses of considerable size from the valves and inner surface of the heart. Not unfrequently, indeed, lumps of old laminated fibrine of even considerable magnitude are found loose in the cavities of the heart, having probably dropped off before death; and sometimes a mass of this kind may be found some distance along the aorta or pulmonary artery.

It is clear, then, that such fibrinous deposits may admit of being very readily detached, and it must be equally clear, that once floating freely in the blood, they are exposed to the almost certain consequence of being transmitted with this fluid, and stopped at the first vessel too narrow to allow of their transit.

The parts of the vascular system within which these tramsmitted masses of fibrine may be found, will, of course, depend, in a great measure, upon whether they proceeded from the right or left side of the heart. Thus, if they have been detached from either the aortic or mitral valves, they will pass into the blood propelled by the left ventricle into the aorta and its subdivisions, and may be arrested in any of the systemic arteries or their ramifications in the various organs, especially those which, like the brain, spleen, and kidneys, receive large supplies of blood directly from the left side of the heart.

If, on the other hand, the fibrinous masses are derived from the pulmonary or tricuspid valves, the pulmonary artery and its subdivisions within the lungs will necessarily become the primary, if not the exclusive seat of their subsequent deposition. A division of the subject being thus naturally formed, I propose to embody the remarks I am about to submit to tho society under two principal heads, considering—

1st. The remote effects resulting from the separation of fibrinous or analogous deposits from the valves or interior of the left side of the heart; and

2d. The corresponding effects produced by the detachment of like deposits from the valves or interior of the right side of the heart.

### PART I.

*On the effects which may result from the separation of fibrinous deposits from the valves or interior of the left side of the heart, and their circulation with the systemic blood.*

In endeavoring to elucidate this part of the subject, I beg to draw attention, in the first place, to instances in which it seems probable that masses of considerable magnitude have been detached from the left side of the heart, and subsequently arrested in an arterial channel of notable size; secondly, to some of the effects which seem to ensue when smaller arterial vessels or capillaries are similarly blocked up; and, thirdly, to circumstances which make it probable that, not unfrequently, the introduction of particles of fibrine into the circulating blood gives rise to constitutional symptoms indicative of a poisoned state of this fluid.

1. The first three cases which I shall offer, are, in many respects, identical; for in each death seemed to ensue from softening of the brain, consequent on obliteration of one of the main cerebral arteries by a mass of fibrinous material, apparently derived directly from warty growths on the left valves of the heart.

Case I.—Margaret Shaw, æt. thirty-four, a pale, weakly-looking woman; admitted into St. Bartholomew's Hospital, under Dr. Roupell, about the middle of July, 1850, on account of pains in her lower limbs, and general debility. A loud systolic murmur was heard all over the cardiac region. No material change ensued in her condition until August 7th, when, while sitting up in bed eating her dinner, she suddenly fell back as if fainting, vomited a little, and when attended to was found speechless, though not unconscious, and partially hemiplegic on the left side. The hemiplegia increased, involving the left side of the face as well as the limbs, and gradually became complete in regard to motion, while sensation seemed to remain unimpaired. She continued speechless and hemiplegic, but without loss of consciousness, for five days, when she quietly died.

On examining the body, six hours after death, the skull and dura mater were found natural; but the small vessels of the pia mater were much congested, the congestion amounting, in some places, almost to ecchymoses. The right corpus striatum was softened to an extreme degree, being reduced to a complete pulp of a dirty greyish-white tint, and without any remains of its characteristic striated structure. The corresponding optic thalamus was healthy; but a condition of pale softening, similar to that effecting the corpus striatum, existed also to a considerable extent in the posterior lope of the right cerebral hemisphere. The rest of the cerebral substance of this hemisphere was soften than natural, and appeared to contain less blood than ordinary. All other parts of the brain were healthy. The right middle cerebral artery just at its commencement was plugged up by a small nodule of firm, whitish, fibrinous-looking substance, which, although not adherent to the walls of the vessel, must have rendered its canal almost, if not quite, impervious. With the exception of a speck or two of yellow deposit in their coats, the rest of the vessels at the base of the brain were healthy and filled with dark blood.

The heart was enlarged; on its exterior, were several broad white patches of old false membrane. The right cavities and left auricle contained recent separated coagula, the fibrine firm and whitish. The right valves were healthy, so also were the aortic, with the exception of slight increase of thickness. The mitral valve was much diseased, the auricular surface of its large cusp being beset with large warty excrescences of adherent blood-stained fibrine. There were a few scattered deposits in the coats of the aorta. The right common iliac artery, about an inch above the origin of its internal branch, was blocked up by a firm, pale, laminated coagulum, which extended into the internal iliac, and for about a quarter of an inch down the external iliac, where it terminated rather abruptly. The lower portion of the coagulum was colorless, and softer and more crumbling than the upper, which was also more blood-stained and laminated. There was no adhesion of the coagulum to the walls of the vessels. No similar clot existed in the iliac vessels on the opposite side. The pleuræ were adherent in places; the lungs œdematous, and in places solidified by compact greyish-white masses, such as might result from uncured pneumonia. The pulmonary vessels were free from old coagula.

The liver and intestinal canal were healthy. The spleen was large, pale, and soft. One large portion, about a fourth of the organ was converted into a mass of firm, yellowish-white, cheesy substance. The kidneys were pale, rough, and granular. Within the cortex of the right were several large masses of yellow deposit, surrounded by patches of redness. The portions of medullary structure passing to these deposits were compact, dryish and yellow.

In the case just narrated, death evidently resulted from softening of a large portion of the ride side of the brain; and the cause of this softening appeared to be an imperfect supply of blood, consequent on the middle cerebral artery of the same side being obstructed by a plug of fibrine within its canal. I am not aware that there has yet been recorded a case in which fatal softening of the brain resulted from a cause like this; therefore, in itself, this case is one of value. That the existence of the fibrinous coagulum within the cerebral artery was the real cause of the changes in the brain, can, I think, scarcely admit of question. The sufficiency of such an obstruction to produce the effects ascribed to it is fully established by the many instances in which disturbance, or complete arrest of function in a part, with subsequent atrophy or disorganization of its tissue, results from any circumstance which materially impedes or entirely cuts off its supply of blood. Examples of this kind at once suggest themselves; such as the weakened and subsequently degenerated heart, when the coronary vessels are diseased by deposits in their coats; the feebleness, atrophy, or even gangrene, ensuing in the whole or part of a limb whose arteries are similarly diseased or obstructed from any other cause; also the impairment of cerebral function and subsequent softening of the tissue of the brain when the cerebral vessels are much diseased. But, perhaps, the best illustration bearing on the case in question is afforded by the results sometimes observed after ligature of one of the common carotid arteries. Such an operation is not unfrequently followed, almost immediately, by giddiness, loss of speech and unconsciousness, which may pass on even to fatal coma. When not thus speedily fatal, hemiplegia of the side opposite to that of the ligatured artery may ensue, and death may occur from that cause at a more remote period, while examination after death discloses a greater or less amount of disorganization, amounting sometimes to gangrene, in the cerebral substance, especially on that side on which the operation was performed.*

"Admitting this explanation of the mischief which ensued in the brain, one is naturally led to inquire into the source of the fibrinous plug found in the middle cerebral artery. The suddenness with which the cerebral symptoms came on made it probable that the blocking up of the vessel was equally sudden, and not the result of a gradual coagulation of blood in this situation. The absence, too, of all appearance of local mischief in the coats of the vessels at the obstructed part, and of general disease of the arterial coats elsewhere, also pointed to some other than a local origin for the clot; and I formed

---

* The whole subject of the effects produced on the cerebral circulation by ligature of the carotid artery has been so thoroughly discussed by Dr. Burrows, in his work on "Disorders of the Cerebral Circulation," (pp. 72–9,) that it seems quite unnecessary to add more to the remarks made on the subject in the next.

the opinion at the time of the examination, that a portion of the fibrinous growths found on the mitral valve had become detached, and then carried with the stream of blood up the carotid artery, until arrested at the angle whence the middle cerebral artery proceeds. This explanation, which fits equally well for the origin of the plug impacted in the common iliac artery, appears so reasonable, that it is difficult to doubt its correctness; for, as already remarked, it is quite conceivable, that portions of the loosely adhering masses of fibrine might be readily washed off by the stream of blood continually passing over the valve; and that when once admitted into the circulating current, such portions would necessarily be arrested the moment they arrived at a vessel too small to allow of their transit along its canal."

The particulars of two other cases very similar to the above are then given. At the conclusion of the narrative of the third case, Dr. Kirkes remarks, "In all essential respects, this case closely resembles the two previously narrated. In each, there was pale softening of the brain, a plug of fibrine obliterating the canal of one of the main cerebral arteries; masses of fibrinous deposit in the kidneys and spleen; and which seemed to be the source of the mischief elsewhere, large, warty, fibrinous excrescences on the left valves of the heart.

"So many, and yet such rare features of resemblance, cannot fail in demonstrating a very close connected between the several morbid appearances so exactly reproduced in each case."

At first, it appears singular, that in each of those cases, as also in others I have had the opportunity of seeing, the clot should be found as nearly as possible in the same situation. But a glance at the arrangement of the arteries at the base of the brain, especially in an injected specimen, will make it clear that this point is, of all others, the one most likely to arrest a solid mass floating in the blood, transmitted to the brain by the internal carotid artery; for almost directly after its entrance into the skull, the carotid divides into its two main branches, the middle and anterior cerebral, which immediately diverge in almost opposite directions. The sudden diminution in size, resulting from the divsion, and the different directions at once taken by the two branches, will together tend to make the angle whence the branches diverge, well calculated to arrest any solid body transmitted along the carotid; while, since of the two branches, the middle cerebral is the largest, and also maintains more nearly than the anterior the original direction of the trunk from which they both sprang, a solid body seems more likely to pass into it than into the anterior division. And such is found to be the case; for if the plug is not found sticking directly at the angle, it is found a short distance up the canal of the middle cerebral.

"It has long been admitted, that any disease of the cerebral arteries sufficient to impede the transit of a due quantity of blood may induce softening of the brain, from imperfect nutrition. But the diseased state of the vessels to which nearly all observations on the subject apply, have reference only to the peculiar fatty or atheromatous condition so frequently presented by the coats of the cerebral arteries, especially in persons of advanced life. And I have been able to meet with very few recorded instances in which distinct fibrinous clots have been noticed blocking up the canal of any of the arteries of the brain; and even when their existence has been noted, the conditions leading to their formation, and the relation which they bear to the attendant cerebral softening, have, so far as I know, never received an explanation similar to that which I now beg to offer."

2d. Dr. Kirkes then proceeds to consider some of the effects which smaller portions, similarly detached and arrested in minute arterial branches or in capillaries, appear capable of producing. One of these effects seems to be displayed by the singular masses of yellow fibrinous-looking substance not uncommonly found in the spleen, kidneys, and other organs, and hitherto described under such names as "capillary phlebitis," "metastases," or "fibrinous deposits."

In 19 cases in which he has observed these deposits, not only were the heart or the valves diseased in each of them, but in 14 of them there were fibrinous growths on the surface of the left valves or interior of the left cavities, while in the remaining 5 there is simple mention of valvular disease, without any statement as to whether fibrinous deposits existed or not. Especial attention is directed to the fact of the co-existence of these minute spots with the large and clearly discernible masses, for since it seems to prove the identity of the two forms of disease, it likewise makes it probable that the small red spots with their yellowish centres, even when found uncombined with the larger masses, have originated in the same cause which led to their formation in other cases.

3d. In regard to the introduction of particles of fibrine into the circulating blood giving rise to phenomena, indicative of a poisoned state of that fluid, he narrates a case, selected from several similar to it, in which the symptoms were very obscure from the beginning, and the obscurity increased as the symptoms of cerebral oppression came on, and proved rapidly fatal. The lad's depressed, languid aspect, with an abundant petechial eruption, seemed to indicate, in the absence of a clear history, the existence of low fever, and the restless, delirious manner in which he passed his first night in the Hospital, favored the view and justified the employment of wine and simple salines; yet the fatal coma which rapidly supervened two days after admission, was quite unintelllgible on this supposition, and could only be understood by an examination after death. The autopsy revealed the existence of friable, loose, fibrinous vegetations on both the mitral and aortic valves. From these, he thinks, portions had probably separated during life, and transmitted by the blood, had been arrested in the capillary net-works and smaller arteries, thus producing the various petechial-looking spots in the skin, and most of the serous and mucous surfaces of the body, the buff colored blotches and streaks, and the solid fibrinous masses in the kidneys and spleen, while they probably caused the fatal issue in the case by the pressure resulting from the extreme engorgement of the minute vessels of the pia mater and the substance of the brain.

### PART II.

*On the effects which may result from the detachment of fibrinous deposits from the right valves of the heart.*

Dr. Kirkes infers also that these deposits may induce corresponding secondary affections of the lungs. It may be clearly shown, he thinks, that most of the fibrinous or other secondary deposits there, also many of the old coagula found in the pulmonary artery or its branches, and possibly some forms of pulmonary apoplexy, are closely connected with, if not actually dependent upon, fibrinous deposits on the valves, or interior of the right side of the heart, or materials transmitted through the heart by venous blood. Cases substantiative of his views are related.

In conclusion, he recapitulates the principal points he has endeavored to establish, which are, 1st: The general fact that fibrinous concretions on the valves or the interior of the heart admit of being readily detached during life, and mingled with the circulating blood; 2dly, That if detached and transmitted in large masses, they may suddenly block up a large artery, and so cut off the supply of blood to an important part; if in smaller masses, they may be arrested in vessels of much less size, and give rise to various morbid appearances in internal organs; while, under other circumstances, the particles mingled with the blood may be extremely minute, possibly the débris of softened fibrine, yet in sufficient quantity and with sufficient power to produce a poisoned state of the circulating fluid, as manifested in the production of typhoid symptoms: 3dly, That the effects produced and the organs affected, will be in great measure determined by the side of the heart from which the fibrinous masses have been detached; for, if the right valves have furnished the source of the fibrine, the lungs will bear the brunt of the secondary mischief, displaying it in coagula in the pulmonary arteries, and various forms of deposit in the pulmonary tissue; but if, as is far more commonly the case, the left valves are affected, the mischief is more widely spread, and may fall on any systemic part, but especially on those organs which, such as the brain, spleen, and kidneys, are largely and directly supplied with blood from the left side of the heart.

**Abstract of article in Medico-Chirurgical Transactions.—Med. Examiner.**

